# A descriptive study to explore working conditions and childcare practices among informal women workers in KwaZulu-Natal, South Africa: identifying opportunities to support childcare for mothers in informal work

**DOI:** 10.1186/s12887-019-1737-7

**Published:** 2019-10-25

**Authors:** Christiane Horwood, Lyn Haskins, Laura Alfers, Zandile Masango-Muzindutsi, Richard Dobson, Nigel Rollins

**Affiliations:** 1 Centre for Rural Health, University of KwaZulu-Natal, Durban, South Africa; 2 WIEGO, Durban, South Africa; 3 Asiye eTafuleni, Durban, South Africa; 4 Department of Maternal, Newborn, Child and Adolescent Health, World Health Organization, Geneva, Switzerland

**Keywords:** Breast feeding, Working women, Workplace health, Child health, South Africa, Informal economy

## Abstract

**Background:**

Although women working in the informal economy are a large and vulnerable group, little is known about infant feeding and childcare practices among these women. The aim of this study was to explore childcare practices among mothers in informal work.

**Methods:**

A cross-sectional survey among mothers with children aged < 2 years working in the informal economy in an urban and a rural site in KwaZulu-Natal, South Africa. Participants were selected using purposive and snowball sampling.

**Results:**

A total of 247 interviews were conducted with 170 informal traders and 77 domestic workers. Most mothers lived with their child (225/247, 91.1%), had initiated breastfeeding (208/247; 84.2%) and many were still breastfeeding (112/247; 45.3%). Among 96 mothers who had stopped breastfeeding, the most common reason was returning to work (34/96; 35.4%). Many mothers relied on family members, particularly grandmothers, to care for their child while they were working (103/247, 41.7%) but some mothers took their child with them to work (70/247; 28.1%). Few fathers participated in the care of their child: 54 mothers (21.9%) reported that the father had ever looked after the child while she was away from home. Domestic workers were less likely than informal traders to take their child to work (*p* = 0.038).

Women reported receiving a salary from an informal employer (119), or being own-account workers (120) or being unpaid/paid in kind (8). Most participants were in stable work (> 4 years) with regular working hours, but received very low pay. Domestic workers were more likely than informal traders to have regular working hours (*p* = 0.004), and to be earning >$240 per month (*p* = 0.003). Mothers reported high levels of food insecurity for themselves and their child: 153 mothers (61.9%) reported having missed a meal in the past month due to lack of resources to buy food, and 88 (35.6%) mothers reported that their child had missed a meal for this reason.

**Conclusion:**

This study provides a preliminary description of informal women workers who, despite having stable work, are vulnerable, low paid and food insecure. These women may require support to provide optimal childcare and nutrition for their children.

## Background

More than half of the global workforce works in the informal economy, which exists in all labour markets in both low and high-income countries [1]. In many parts of the world most new jobs created are in informal work, and it is now recognised that the informal economy does not necessarily recede as economies grow, with many successful economies showing increases in informal employment [2]. The size, heterogeneity and universality of the informal economy, its ability to emerge in new guises in new situations, and its contribution to gross domestic product (GDP) has led to increasing recognition of its importance [2]. Informal work can be defined in different ways but in all settings and definitions, informal workers generally lack the social protection enjoyed by formal workers, including access to sick leave, maternity leave or unemployment benefits. The informal work environment is characterised by low earnings, unsafe working conditions, and poor job security [3]; as a result, working in the informal economy is frequently associated with poverty and vulnerability. Women are disproportionally represented in the informal economy and childcare responsibilities fall disproportionally on women, so it is challenging to manage these dual responsibilities, and the need to work impacts on how these mothers care for and feed their children [4].

In South Africa (SA) in 2017, of a working population of 16 million people, almost 5 million were in informal work. Among 6.8 million working women, almost 2 million were working informally, the largest groups being informal traders, agricultural workers and workers in private homes (domestic workers). Almost all domestic workers are women, accounting for around 50% of all women informal workers [5]. Two-thirds of informal workers are employed, whereby they receive payment from an employer in an informal enterprise [6]. However, informal workers may also be own-account workers (25%) or informal employers themselves (3%). Overall, in the informal work environment in SA, women earn around 25% less income than men [7].

Providing children with an adequate diet is essential for child health and development, and for children of low income mothers working informally, is crucial to breaking the cycle of poverty. In particular, optimal breastfeeding practices, which include exclusive breastfeeding (EBF) for 6 months and sustained breastfeeding to 2 years, are critical for child health and development [8, 9]. It is estimated that high coverage (> 90%) of optimal breast feeding practices could prevent over 800,000 child deaths globally [10], and the United Nations has included achieving 50% EBF in the first 6 months, as a key target to eradicate malnutrition [11].

However, early return to paid employment is consistently associated with shorter durations of breastfeeding in formal work settings [12–15]. Several workplace interventions have been shown to improve breastfeeding rates among working women in the formal work sector [16–20]. Longer durations of maternity leave can improve breastfeeding practices, as can providing on-site childcare [13, 21, 22]. However, breastfeeding remains a challenge for working women and, even in formal settings, mothers who choose to breastfeed frequently suffer greater and more prolonged earnings losses compared to women who choose not to breastfeed [23].

Achieving optimal breastfeeding has particular challenges for informal women workers. Women working as street vendors, for example, face spatial challenges of working in a public space that may change from day-to-day, and where hygiene and safety conditions are often poor. As a result, mothers describe the difficulties of balancing the economic necessity of working, often returning to work soon after the baby is born, with continuing to breastfeed, particularly given the lack of a supportive environment in many low paid or informal jobs [4, 15, 24]. In contrast, supportive work environments in some low-income or informal work settings have allowed working mothers to keep their infants close to their work stations, helping them to successfully breastfeed [25]. Some studies have shown that mothers working informally are more likely to breastfeed compared those in formal employment, suggesting that the informal work environment, by its flexible nature, may be more supportive to breastfeeding [26, 27], and mothers may chose informal work because it allows them to adapt working hours to suit childcare [4].

Appropriate interventions to support breastfeeding and childcare must strike a balance between the woman’s right to work and provide for herself and her family, and the rights of children to the best care to promote health and development. Given the large numbers of women in informal work, its association with vulnerability, and the conditions and quality of informal employment, it is important to establish how these forms of work impact on childcare practices. The aim of this study was to understand and explore possible factors impacting on child care practices among women informal workers, including income, food security, work environment, household/childcare environment, and access to health services.

## Methods

A cross sectional survey was conducted among informal women workers with children aged < 2 years, in two sites in the province of KwaZulu-Natal (KZN), SA to explore childcare and feeding practices, income, working environment, among mothers in different types of informal work. Two main sectors of the informal economy were included, these were domestic workers, who make up the largest share of women’s informal employment in SA, and informal traders. Informal traders included street traders, and traders working in formal markets with some infrastructure provided, including those who are cooking and selling food in the street or market.

### Definitions

For the purposes of the study, women working in the informal economy were defined as women working as employers or employees in an informal business that is not registered for VAT or income tax, or who were own-account (self-employed) workers. Informal workers, by definition, did not make contributions, either by themselves or through their employer, to UIF (unemployment insurance), and did not have a contract of employment. Domestic workers were defined separately as unskilled workers working in private households doing domestic or childcare work, regardless of whether they had contracts or made UIF contributions.

### Study sites

The urban study site, where urban informal traders were recruited, is one of the largest informal trading areas in Durban, is close to a busy transport route and has a population of approximately 6000-8000 traders, most of whom are women. This market has organised systems and functional infrastructure for informal workers. Many traders in this area are recognised by the local municipality and pay a fee to the municipality for use of the space, but others work on the streets in the same area.

The rural site, selected to include rural and peri-urban informal traders, was in a predominantly rural district with three small to medium sized towns, where there were both covered markets and street markets. We recruited street traders in market areas of each of the three small towns. Although informal work is common in this area, the infrastructure available to informal workers is less organised compared to the urban site.

Domestic workers were recruited in the residential areas of same urban city and small rural towns.

### Inclusion and exclusion criteria

Determination of eligibility for participation was guided by a structured screening tool based on the definitions of informal work outlined above. Participants were eligible to participate if they were aged 18 years or older, had been in informal work for at least 3 months, were working at least 10 h per week, and the mother of a living child under the age of 2 years. Participants were excluded if they were unable to communicate in a local language with the researchers.

### Sampling

Sampling used a non-probability approach, and did not aim to be representative of all informal workers in the study sites. Sampling used a combination of purposive and snowball sampling techniques to recruit informal traders and domestic workers. In the urban site, informal traders were identified and recruited with the assistance of a local NGO that works with the traders (17). In the rural site, study staff approached local community leaders in all three small towns and received permission to approach informal workers directly.

Domestic workers were sampled separately to ensure that this group was represented. Field workers approached domestic workers in areas where they are observed to congregate, including outside schools, on street corners and at bus stops. Domestic workers were also requested to identify other eligible domestic workers with young children using a snowball sampling method.

### Data collection

A survey questionnaire was developed which included: mothers details; household information; childcare practices and feeding; knowledge and attitudes about breastfeeding; mothers work environment; mothers income, household food security and access to health services. These focus areas were identified based on the literature review, and were guided by the UNICEF framework for the causes of malnutrition and inadequate childcare as presented by Black et al. [28]. Questions related to food security were taken from the USAID household food insecurity access scale [29].

The tool was translated into isiZulu and was piloted in informal settings away from the study sites, and adapted accordingly.

Structured interviews were conducted by trained fieldworkers in the local language (isiZulu) or in English, according to the ethnic group and preference of the participants.

### Data analysis

Data gathered from the questionnaire was entered into SPSS version 24 and analysis was done in Stata V13. Descriptive statistics and frequencies were calculated. Sub-group comparisons were done using chi-squared tests. Chi-squared Fishers Exact Test was used as appropriate. *P* value< 0.05 were considered to be statistically significant.

## Results

A total of 247 interviews were conducted with informally working mothers between February and May 2017. Interviews were conducted with 170 informal traders and 77 domestic workers in the two sites: 122 mothers participated in the urban site and 125 mothers were from the rural district.

The mean age of participating mothers was 32.2 years (SD 6.7). Demographic characteristics were largely similar between domestic workers and informal traders. However, there were significant differences in the household environment between the two groups with domestic workers having better access to safe water and sanitation in their homes compared to informal traders (Table 1).

**Table 1:** Demographic characteristics of participating mothers and children

	All Mothers *N* = 247 (%)	Domestic Workers *N* = 77 (%)	Informal traders *N* = 170 (%)	*P* value
Mothers age category				
18-25 years	54 (21.9)	10 (13.0)	44 (25.9)	**0.03**
26-35 years	103 (41.7)	30 (39.0)	73 (42.9)	0.89
36-45 years	88 (35.6)	36 (46.8)	52 (30.6)	**0.015**
46 and over	2 (0.8)	1 (1.3)	1 (0.6)	
Mothers education				
No schooling	8 (3.2)	3 (3.9)	5 (2.9)	0.38
Primary school Grade 1-7	45 (18.2)	11 (14.3)	34 (20.0)	0.28
Secondary school Grade 8-11	130 (52.6)	40 (51.9)	90 (52.9)	0.89
Completed school	64 (25.9)	23 (29.9)	41 (24.1)	0.34
Mothers current relationship status				
Married	36 (14.6)	5 (6.5)	31 (18.2)	**0.015**
Separated /divorced / Widowed	1 (0.4)	1 (1.3)	0	
In a relationship, living with partner	39 (15.8)	13 (16.9)	26 (15.3)	0.75
In a relationship, not living with partner	120 (48.6)	43 (55.8)	77 (45.3)	0.12
Single	51 (20.6)	15 (19.5)	36 (21.2)	0.82
Child characteristics: Age group of the child				
0-6 months	54 (21.9)	16 (20.8)	38 (22.4)	0.78
7-11 months	58 (23.5)	21 (27.3)	37 (21.8)	0.34
12-18 months	77 (31.2)	22 (28.6)	55 (32.4)	0.40
18 months and more	58 (23.5)	18 (23.4)	40 (23.5)	0.98
Female	141 (57.1)	43 (55.8)	98 (57.6)	0.75
Child lives with mother	225 (90.7)	66 (85.7)	159 (92.9)	**0.046**
Any biological child has died	52 (21.1)	16 (20.8)	36 (21.1)	0.94
HOUSEHOLD characteristics: main source of drinking water in the home				
Piped -inside the home	91 (36.8)	39 (50.6)	52 (30.6)	**0.002**
Piped – outside the yard	70 (28.3)	19 (24.7)	51 (30.0)	0.39
Piped – public tap	69 (27.9)	19 (24.7)	50 (29.4)	0.44
River/dam/lake/rainwater/ borehole/ tanker	17 (6.9)	0	17 (10.0)	
Type of toilet used by household				
Flush toilet inside	93 (37.7)	43 (55.8)	50 (29.4)	**< 0.001**
Flush toilet outside	41 (16.6)	17 (22.1)	24 (14.1)	0.12
Ventilated pit latrine	46 (18.6)	9 (11.7)	37 (21.8)	0.06
Pit latrine	62 (25.1)	8 (10.4)	54 (31.8)	**< 0.001**
Bucket toilet/Bush / veld / no toilet	5 (2.0)	0	5 (2.0)	
Household connected to electricity	225 (91.1)	76 (98.7)	149 (87.6)	
Main source of fuel used for cooking				
Electricity	197 (79.8)	72 (93.5)	125 (73.5)	**< 0.001**
Gas	6 (2.4)	1 (1.3)	5 (2.9)	0.44
Wood	31 (12.6)	3 (3.9)	28 (16.5)	**0.006**
Paraffin	13 (5.3)	1 (1.3)	12 (7.1)	0.06

data in bold represents P < 0.05

### Feeding practices

Reported feeding practices among participating mothers and mothers’ perceptions of breastfeeding in the workplace are shown in Table 2. Informal traders were significantly more likely to be currently breastfeeding compared to domestic workers. Domestic workers were significantly more likely to report feeling comfortable taking the baby to work and expressing breastmilk while at work. Most mothers had a good knowledge of the importance of breastfeeding and of breastfeeding practices, and reported positive attitudes towards breastfeeding but some knowledge gaps were identified (Table 3).

**Table 2:** Feeding practices of informally working mothers

	All Mothers *N* = 247 (%)	Domestic Workers *N* = 77 (%)	Informal traders *N* = 170 (%)	*P* value
Mother initiated breastfeeding	208 (84.2)	68 (88.3)	140 (82.4)	0.23
Mother has given the baby expressed breastmilk at any time	76 (30.7)	29 (37.6)	47 (27.6)	0.11
Mother reports ever having taken the baby to work with her	130 (52.6%)	36 (46.7%)	94 (55.3%)	0.21
Mothers’ perceptions of breastfeeding in the workplace				
Mother reports she would feel comfortable taking her child to work	101 (40.9%)	43 (55.8%)	58 (34.1%)	**0.001**
Mother reports she would be able to breastfeed while at work	121 (49.0%)	40 (51.9%)	81 (47.6%)	0.53
Mother reports she would be able to express breastmilk while at work	61 (24.7%)	30 (39.0%)	31 (18.2%)	**0.005**
Current feeding practice				
Currently breastfeeding	112 (45.3)	26 (33.8)	86 (50.6)	**0.01**
Stopped breastfeeding	96 (46.1)	42 (61.8)	54 (38.1)	**0.001**
Never breastfed	39 (15.8)	9 (11.7)	30 (17.6)	0.265
Main reason for stopping breastfeeding (mothers who have stopped breastfeeding)				
	*N* = 96	*N* = 42	*N* = 54	
Experiences of breastfeeding	11 (9.3)	4 (9.5)	7 (13.0)	0.75
Had to go back to work /be away from the baby	34 (35.1)	17 (40.5)	17 (31.5)	0.36
Mothers health including HIV infection	14 (14.4)	7 (16.7)	7 (13.0)	0.61
Advised to stop breastfeeding (by health worker or family member)	8 (8.3)	5 (6.5)	3 (5.3)	0.19
Other reason	29 (30.2)	9 (21.4)	20 (37.0)	0.14

data in bold represents P < 0.05

**Table 3:** Knowledge and attitudes of mothers about breastfeeding

	Correct	Mothers giving correct answer *N* = 247 (%)
For how long should you breastfeed?	2 years	188 (76.1)
How old should the baby be when you start giving other food and fluids?	6 months	216 (87.4)
Initial breast production of yellow water (colostrum) is nutritionally useless for the baby and should be discarded	False	155 (62.8)
Breastfeed babies have less diarrhoea	True	233 (94.3)
Infant formula contains all the ingredients found in human breastmilk	False	222 (89.9)
All babies should be given water between feeds during summer	False	43 (17.4)
A mother who feels that the baby is not getting enough breastmilk should top up feeds with formula milk	False	142 (57.5)
Babies who cry a lot should be given a pacifier / dummy	False	219 (88.7)
Breastfeeding is easier than feeding with infant formula	True	231 (93.5)
When you go back to work it is better to stop breastfeeding completely rather than mixed feed	False	56 (22.7)
It is better for the health of the child to give a small amount of breastmilk rather than no breastmilk at all	True	224 (90.7)

### Child care

The majority of women stopped working after the child’s birth for at least 2 months (Table 4).

**Table 4:** Current childcare practices

	All Mothers *N* = 247 (%)	Domestic Workers *N* = 77 (%)	Informal traders *N* = 170 (%)
Mother was working when child was born?	198 (80.2)	65 (84.4)	133 (78.2)
Mothers working when child was born:	*N* = 198 (%)	*N* = 65 (%)	*N* = 133 (%)
Working in their current job at time of birth	186 (93.4)	58 (89.2)	128 (96.2)
Mother took time off when the baby was born	174 (87.8)	58 (89.2)	116 (87.2)
Means of support among mothers who took time off when the baby was born:	*N* = 174(%)	*N* = 58 (%)	*N* = 116 (%)
Child support grant	84 (34.0)	32 (41.6)	52 (30.6)
Father supported	66 (26.7)	26 (33.8)	40 (23.5)
Other family member supported	46 (18.6)	14 (18.2)	32 (18.8)
Savings	95 (38.5)	38 (49.4)	57 (33.5)
Other	21 (8.5)	10 (13.0)	11 (6.5)
Age of the child when the mother returned to work			
Not working at the time of birth	49 (19.8)	12 (15.6)	37 (21.8)
Did not take any time off	24 (9.7)	7 (9.1)	17 (10.0)
Less than 1 month	4 (1.6)	1 (1.3)	3 (1.8)
1 to < 2 months	18 (7.3)	3 (3.9)	15 (8.8)
2 to < 3 months	37 (15.0)	10 (13.0)	27 (15.9)
3 to < 4 months	60 (24.3)	26 (33.8)	34 (20.0)
4 to < 5 months	32 (13.0)	11 (14.3)	21 (12.4)
5 to < 6 months	10 (4.1)	5 (6.5)	5 (2.9)
More than 6 months	11 (4.5)	1 (1.3)	10 (5.9)
All Mothers			
Person who usually cares for the child while mother is working			
Childs grandmother	65 (26.3)	16 (20.8)	49 (28.8)
Childs father	1 (.4)	1 (1.3)	0
Childs sibling	8 (3.2)	2 (2.6)	6 (3.5)
Other relative	29 (11.7)	13 (16.9)	16 (9.4)
Non relative	52 (21.1)	19 (24.7)	33 (19.4)
Mother herself (takes child to work / works at home)	70 (28.3)	15 (19.4)	55 (32.4)
Child not living with the mother	22 (8.9)	11 (14.3)	11 (6.5)
Place where child is cared for while mother is working			
At mothers current residence	98 (39.7)	28 (36.4)	70 (41.2)
At the carers home	35 (14.2)	13 (16.9)	22 (12.9)
At a crèche or school	22 (8.9)	10 (13.0)	12 (7.1)
Mother takes child to work	70 (28.3)	15 (19.5)	55 (32.4)
Mother pays for childcare	71 (28.7)	29 (37.7)	42 (24.7)
Water source at the place where the child is cared for			
Piped – inside the house or yard	102 (41.3)	37 (48.1)	65 (38.2)
Piped – outside the yard	41 (16.6)	13 (16.9)	28 (16.5)
Other	12 (4.9)	1 (1.3)	11 (6.5)
Do not know	1 (0.4)	0	1 (0.6)
Brings child to work; water available	82 (33.2)	26 (33.8)	56 (32.9)
Brings child to work; water not available	9 (3.6)	0	9 (5.3)

Most mothers relied on family members to take care of the child while they were working. Among 89 carers who were neither a parent nor a grandmother, there were 37 carers under the age of 18 years. Childcare practices were similar between informal traders and domestic workers, however domestic workers were significantly less likely to take their child to work with them (*p* = 0.038), and were significantly more likely to pay for childcare (*p* = 0.037).

### Role of the father in childcare

Among 247 participating mothers, 15 (6.1%) mothers reported that the father of the child was dead or his whereabouts were unknown, 171 (69.2%) were currently in a relationship with the child’s father, and 72 (29.1%) mothers were staying in the same house as the child’s father. Among fathers whose whereabouts were known, most were working (158/232; 68.1%): 75 fathers were in formal employment and 80 fathers were informal workers. Fathers usually saw their children regularly: 138/232 (59.5%) at least weekly; 30/232 (12.9%) at least monthly, but some fathers saw their child less than once per month or never (48/232; 20.7%). However, few fathers participated directly in the care of the child: 54 mothers (21.9%) reported that the father had ever looked after the child while she was away from home, and 41 (16.6%) that the father had ever attended the clinic with the child.

### Working conditions

Table 5 shows the working conditions of domestic workers and informal traders. Domestic workers were more likely to have regular working hours (*p* = 0.004) and to be working indoors (*p* < 0.001) and to be earning > R3000 ($200) per month (*p* = 0.003). In contrast informal traders were more likely to be working 6-7 days per week (p < 0.001) and to be unpaid or paid in kind (*p* = 0.006). There were no other significant differences shown between working conditions of domestic workers and informal traders.

**Table 5:** Working conditions of participating mothers

	All workers *N* = 247 (%)	Domestic workers *N* = 77 (%)	Informal traders *N* = 170 (%)
Main type of work			
Domestic Worker	77 (31.2)	77 (100)	0
Street/ informal vendor (non-cooking)	91 (36.8)	0	91 (53.5)
Street/ informal vendor cooking	36 (14.6)	0	36 (21.2)
Market trader	39 (15.8)	0	39 (22.9)
Waste picker	8 (3.2)	0	8 (4.7)
Home-based worker	6 (2.4)	0	6 (3.5)
Hairdresser	1 (0.4)	0	1 (0.6)
Has more than one job	11 (4.4)	0	11 (6.5)
Has regular working hours	164 (66.4)	61 (79.2)	103 (60.6)
Works in the same location every day	212 (85.8)	71 (92.2)	141 (82.9)
Duration working in current position			
Less than a year	42 (17.0)	12 (15.6)	30 (17.6)
1-3 years	79 (32.0)	32 (41.6)	47 (27.6)
4 or more years	126 (51.0)	33 (42.9)	93 (54.7)
Days per week worked			
1-3 days	21 (8.5)	9 (11.7)	12 (7.1)
4-5 days	55 (22.3)	32 (41.6)	23 (13.5)
6-7 days	171 (69.2)	36 (46.8)	135 (79.4)
Workplace			
Own home	8 (3.2)	0	8 (4.7)
Own premises	41 (16.6)	1 (1.3)	40 (23.5)
Employer premises	93 (37.7)	76 (98.7)	17 (10.0)
Temporary structure	30 (12.1)	0	30 (17.6)
Fixed or temporary stall in a market	25 (10.1)	0	25 (14.7)
Street stall/vehicle/cart, goods on ground	36 (14.6)	0	36 (21.2)
Landfill site or dump site	6 (2.4)	0	6 (3.5)
No fixed location / mobile	7 (2.8)	0	7 (4.1)
Construction site	1 (0.4)	0	1 (0.6)
Indoor or outdoor work			
Mostly inside	112 (45.3)	76 (98.7)	36 (21.2)
Mostly outside with shelter	50 (20.2)	1 (1.3)	49 (28.8)
Mostly outside without shelter	85 (34.4)	0	85 (50.0)
Job type			
Own account worker	120 (48.6)	0	120 (70.6)
Employer (informal business)	1 (0.4)	0	1 (0.6)
Employee (informal business)	126 (51.0)	77 (100)	49 (28.8)
Money paid per month (excludes own account workers)	*N* = 126	*N* = 77	*N* = 49
Do not receive any money (unpaid)	8 (6.4)	1 (1.3)	7 (14.3)
Less than R1000.00 (<$70)	25 (19.8)	10 (13.0)	15 (30.6)
Between R1000.00 and R3000.00 ($80-240)	81 (64.3)	54 (70.1)	27 (55.1)
More than R3000.00 (>$240)	12 (9.5)	12 (15.6)	0 (0.0)

There were 118 workers who received payment from an employer, including almost all (76/77) domestic workers and a minority of informal traders (42/170). Most paid workers reported that they received a fixed salary (112/ 118; 94.9%), and that their salary was paid according to a rate that was either hourly (2/118; 1.7%), daily (24/118; 20.3%), weekly (18/118; 15.3%) or monthly (68/119; 57.1%). Some mothers reported that they had received pay when they were unable to work because they were sick (40/118; 33.9%). In this sub-group of paid employees, domestic workers were more likely than informal traders to be paid monthly (*p* < 0.001) and were more likely to receive sick pay (*p* = 0.02).

Most mothers (209/247; 84.6%) received a child support grant for this child (a government grant of approximately R410 ($25) per child per month), and reported that the father had contributed, either with money or goods, for the child’s upkeep during the past 1 month (148/247; 59.9%). These findings were similar among domestic workers and informal traders.

Mothers reported significant food insecurity for themselves and for their children. Among all participating mothers, 153/247 (61.9%) mothers reported that in the past 4 weeks she had to miss a meal because of lack of resources to buy food, and 39 (15.8%) mothers reported this had happened frequently (more than 10 times in 4 weeks). Similarly, 88 (35.6%) mothers reported that the child had missed a meal due to lack of resources to buy food, and 21 (8.5%) mothers mentioned that this had happened frequently. Food insecurity was similar among informal traders and domestic workers.

### Maternal and child health

Attendance at antenatal clinic (ANC) during the mother’s most recent pregnancy was almost universal (245/247: 99.2%), and most mothers had attended ANC at least four times (216/247: 87.4%) and delivered their baby in a health facility (231/247; 93.5%). Among 240 (97.2%) mothers who reported having had an HIV test, 108 (45.0%) reported testing HIV positive. All HIV positive mothers were currently taking antiretroviral treatment, and 106/108 HIV exposed children had a PCR test.

There were 77 mothers who reported their child had suffered an illness during the past 1 month, and most reported having sought help from a health facility or clinic (58/77; 75.3%). When asked about the child’s most recent clinic attendance, most mothers had taken the child to the clinic themselves (187/247; 75.7%) either taking time off work (136/247; 55.1%) or outside of working hours (51/247; 20.6%). Otherwise the child was taken to the clinic by another family member (56/247; 22.7%), or a non-relative (3/247; 1.2%).

## Discussion

Our findings provide an initial picture of the work and childcare circumstances among women working as informal traders and domestic workers in KZN, SA. Findings suggest that most women working in the informal economy in these sectors have worked in the same job for several years, receive a regular salary either weekly or monthly, and work in the same place each day. However, these women work long hours, with many women earning <R1000 ($80) per month, most do not receive payment when they are sick, and many women work outdoors without shelter. Women report high levels of food insecurity not just for themselves but also for their child. Although working mothers and their children are vulnerable, the relative stability of the work environment provides opportunities for interventions to support childcare and feeding.

Another opportunity for possible interventions is that most children in this age group live with their mother rather than being cared for in a different household, which is a common practice in South African communities. Mothers who do not take the child to work usually rely on family members to look after their children in their own homes, and few mothers paid for childcare. Most carers were grandmothers, and research suggests that grandmothers have a strong role to play in decision making about childcare, infant feeding and child health in several African settings [20–22]. A holistic approach to encouraging breastfeeding among working women should therefore involve the carers who feed the child during working hours, and family members who influence decision-making about childcare. Interventions to improve feeding practices should therefore not only be situated in the workplace but community-based support for childcare could play an important role, and the support of families and communities is likely to be important for success of any proposed intervention.

A range of interventions in the work environment have shown to successfully support breastfeeding in the formal work sector. These include educating mothers about how to continue breastfeeding while away from their child, and providing physical facilities for lactating mothers, including for expressing and storing breastmilk [16]. Mother-friendly work places can effectively support breastfeeding, by providing breastfeeding breaks, flexible working hours, and facilities for the child to stay with the mother safely in a work environment that may otherwise be hazardous [17–20]. Many of these interventions may be adaptable to informal work settings, and employers and/or co-workers could be an important resource to support mothers to breastfeed safely by assisting to provide the time, space and opportunity for mothers to care for their children during working hours.

In addition, it has been suggested that breastfeeding rates could be higher among informal workers because of the flexible nature of informal work, and that working mothers may even choose informal work for this reason [26, 27]. However, a recent study of breastfeeding practices in KZN reported 90.0% of mothers of children aged 14 weeks initiated breastfeeding [30], compared to 84.2% of mothers in this study. This suggests that in our setting breastfeeding is not higher among informal workers, but further research is needed to confirm this.

In the two sectors included in this study, domestic workers appeared to work in better circumstances than informal traders because of the nature of their work. Domestic workers frequently lived in relatively well-resourced situations, often in the home of their employer, and were generally better paid, more likely to receive sick pay, and to work indoors, compared to informal traders. However, despite this, domestic workers were significantly less likely to be taking their child to work, suggesting that they may be unable to take their child to the employers’ household during working hours. This highlights the diverse and unpredictable nature of informal work, so that informally working women in sectors not included in this preliminary study, for example workers in informal shops or factories, or working at home making crafts, are likely to have different childcare challenges. A variety of different approaches will be required to support informal women workers and their children and proposed interventions should be flexible and adaptable to different work settings.

Access to health services was good in this population and similar to the general population of mothers in SA [18]. Mothers usually take the child to the health facility themselves, despite this being associated with possible loss of earnings, providing an opportunity for health workers (HWs) to give mothers appropriate and relevant support for infant feeding throughout the perinatal period. Research suggests that support or advice from HWs can improve breastfeeding practices [19], but it is important that HWs be aware of the particular challenges and vulnerabilities of women in informal work and provide infant feeding counselling relevant to the context in which these mothers find themselves. In particular, HWs require good practical information about maintaining breastfeeding while away from the child, and the challenges associated with achieving ongoing breastfeeding while working. Availability of good quality and relevant counselling could make giving expressed breastmilk to their child while at work a realistic option for many women. A substantial minority of our participants reported that they had given expressed breastmilk to their child, suggesting this practice is familiar to this community, and could be more widely supported and practiced.

A major limitation of this study is that it is not representative of all informal traders or domestic workers. This population is difficult to reach and there was no available sampling frame, so for this formative work we employed a non-probability sampling approach. This almost certainly led to bias towards selecting those sub-sectors of this community that were most visible and easily accessed. Women working at home or employed in other informal settings were excluded, in particular agricultural workers, and it is likely that other sectors have specific challenges. Comparisons between domestic workers and informal traders should be viewed with caution given that these populations were not representative of these sectors. However, our study provides useful preliminary work to inform a future research agenda. In particular, additional research is required to explore child care practices among working women in different informal work sectors and to identify possible interventions. It would be particularly important to employ qualitative methodologies to further explore the interactions between child care and the informal work environment, and to further understand what determines mothers’ childcare practices. Any proposed interventions should be carefully piloted and evaluated in different settings.

## Conclusion

This study provides a preliminary description of this population of women who despite having stable work, are vulnerable, low paid and food insecure. Some opportunities for supporting these mothers to improve feeding practices have been identified from our results, and further research is required to develop and evaluate such interventions. However, any intervention should focus not just on the work setting but on the wider context of the mother’s life, including her home and community, and the role the health system can play in supporting the intervention. Investments in promoting breastfeeding will reap benefits in improved health and development of the children in these communities as well as improving the health of mothers.

## Data Availability

The dataset supporting the conclusions of this article is available on reasonable request made directly to the principle investigator and first author of this study.

## Abbreviations

GDP: Gross Domestic Product
HW: Health worker
IQR: Inter quartile range
KZN: KwaZulu-Natal
NGO: Non-governmental organisation
SA: South Africa
Stats SA: Statistics South Africa
UKZN: University of KwaZulu-Natal
UN: United Nations
VAT: Value added tax
WHO: World Health Organization
